# Evaluating Self-Efficacy among Patients Undergoing Dialysis Therapy

**DOI:** 10.3390/nursrep11010019

**Published:** 2021-03-23

**Authors:** Hayfa Almutary, Nahla Tayyib

**Affiliations:** 1 Medical/Surgical Nursing Department, King Abdulaziz University, Jeddah 21589, Saudi Arabia; 2 Medical/Surgical Department, Faculty of Nursing, King Abdulaziz University, Jeddah 21589, Saudi Arabia; 3 Nursing Practice Department, College of Nursing, Umm Al-Qura University, Alalziziah Makkah 77207, Saudi Arabia; natayyib@uqu.edu.sa

**Keywords:** chronic kidney disease, hemodialysis, peritoneal dialysis, self-efficacy

## Abstract

(1) Background: Perceived disease-related self-efficacy is considered a fundamental component of the successful self-management of chronic diseases. Prior studies have found that self-efficacy is associated with improvements in health behaviors and health status among people with chronic kidney disease (CKD). However, few studies have evaluated self-efficacy among patients undergoing dialysis. (2) Methods: This study was performed to evaluate CKD patients’ self-efficacy and to determine the factors that significantly affect self-efficacy among dialysis patients. This was a cross-sectional study using a convenience sample of 190 patients undergoing dialysis. The patients’ self-efficacy was measured using the CKD Self-Efficacy Scale. Inferential statistics were used to analyze the data. (3) Results: The mean age of the participants was 49.24 ± 13.15 years. Almost half of them were males (48.4%), and 75.3% were married. The majority of the patients (83%) were undergoing hemodialysis. The total score for self-efficacy was 192.57 ± 39.23. Only occupational status and the type of dialysis were significantly and positively correlated with patients’ perceived self-efficacy scores. (4) Conclusions: This study provides primary evidence of the perceived self-efficacy among CKD patients who are on dialysis. The results of this cross-sectional study showed that greater self-efficacy was associated with employment and peritoneal dialysis. Strategies to enhance self-efficacy among dialysis patients, especially those on hemodialysis, are needed.

## 1. Introduction

Chronic kidney disease (CKD) is a global public health problem with increasing incidence, prevalence, and mortality rates [1,2]. The global prevalence of all stages of CKD is currently 9.1% [1]. According to the World Health Organization report, there will be a projected increase of 14% in the mortality rate related to CKD by 2030, accounting for 11.5 million deaths globally [3]. In Saudi Arabia, CKD is the fourth leading cause of death [4]. In recent decades, the number of people who have initiated renal replacement therapy in Saudi Arabia has been increasing rapidly, with a 7.7% annual growth rate of the population on dialysis [5].

CKD can progress to end-stage kidney disease (ESKD) and cause other complications that require dialysis or kidney transplantation [6,7]. Hemodialysis is the most common treatment option used worldwide for the management of ESKD, followed by peritoneal dialysis [8]. Patients undergoing dialysis may experience adverse events, which can have devastating and costly impacts on patient quality of life and healthcare systems [9,10]. Due to the chronic nature of the disease, CKD requires continuous monitoring and care. All healthcare professionals, especially nurses, play key roles in providing care and education to patients and need to empower patients to be actively engaged in the self-management of their disease.

CKD management mainly depends upon the patient’s self-management skills, which include the ability to implement lifestyle modifications and cope with symptoms, medications, and the physical and psychosocial side effects of having CKD and related comorbidities [11]. Patient self-management has five dimensions: communication, partnership in care, self-care activities, self-integration, and adherence to the recommended treatment [11,12].

Perceived disease-related self-efficacy (DSE) is a fundamental component of the effective self-management of chronic diseases [11,13,14]. It is conceptualized as patients’ confidence in their ability to overcome barriers and achieve the desired outcomes [15,16]. According to self-efficacy theory, if individuals believe that they can achieve a result, they will be a more active participant in the management of their condition, which will facilitate adequate disease control [15]. Studies have shown that self-efficacy is a predictor of health behavior improvements, such as adherence to the recommended treatment, communication, problem solving, and social support, among chronic disease patients in general [12,17,18].

Self-efficacy plays a vital role in delaying CKD progression, and it has been shown that a higher level of self-efficacy in CKD patients is associated with a better quality of life [11,19] and lower levels of anxiety and depression [20]. However, there are many factors that could influence CKD patients’ behavior, such as age, sex, type of dialysis, and duration of dialysis [13].

The prevalence of CKD has increased, imposing a burden on the global health care system, and different studies have been shown a positive association between perceived self-efficacy and health care utilization among the CKD population [13,14,21]. Knowing the level of self-efficacy among the CKD population is essential to providing appropriate interventions that promote self-competence with regard to controlling disease progression. In Saudi Arabia, this is the first study to evaluate CKD patients’ self-efficacy and determine the associated factors among dialysis patients. 

## 2. Materials and Methods

### 2.1. Study Design

This study used a descriptive cross-sectional design. 

### 2.2. Sample and Setting 

In this study, we enrolled a convenience sample of patients undergoing dialysis at two dialysis centers in the Western Region of Saudi Arabia. The inclusion criteria were as follows: age ≥ 18 years, dialysis for at least 6 months, able to understand Arabic, and willing to participate in the study. The exclusion criteria were major psychiatric disorders or cognitive impairment, physical dependency, and serious illness. The study was explained verbally and in writing to eligible participants. The sample size was calculated using G*power 3.1 software which is recommended when conducting an observational study. It was used by assuming 95% power (1-beta = 0.95), a type 1 error rate (alpha) of 0.05 (two-tailed), and a large effect size (Cohen’s d = 0.1). Thus, at least 133 participants were needed for this analysis. 

### 2.3. Measures

Demographic and clinical characteristics were collected. These included age, sex, marital status, education level, occupational status, type of dialysis, duration of dialysis, body mass index (BMI), and blood pressure.

Patients’ perceived self-efficacy regarding the management of CKD was assessed using the chronic kidney disease self-efficacy (CKD-SE) scale [11]. This scale consists of 25 items with four subscales (autonomy, self-integration, problem-solving, and seeking social support). The scale is from 0 to 10 points, and a larger number indicates a higher level of confidence regarding the management activity. The total score on the scale ranges between 25 and 250. Furthermore, the total score can be classified into three categories: low self-efficacy (score less than 30), moderate (scores between 30 to 70), and high self-efficacy (score more than 70) [11].

The validity and reliability of the English version of the CKD-SE scale were demonstrated previously in which Cronbach’s alpha coefficients for the subscales ranged between 0.843 and 0.901 [11]. However, the scale was not available in Arabic and needed to be translated. Therefore, we translated the CKD-SE scale into Arabic according to the Brislin back-translation method [22]. First, a certified translator was invited to translate the CKD-SE scale from English into Arabic. Then, an expert panel, which involved renal nurses, nephrologists, and social workers, was consulted to ensure the clarity of the translated version, identify any linguistic mistakes, and maintain cross-cultural equivalence. The input of the expert panel substantively improved the translated version. Then, back-translation from the Arabic version of the CKD-SE into English was performed by a bilingual translator who had not had access to the original version. The original English version and the back-translated version were compared by the researchers. Comparing both versions revealed that there were no differences in meaning between them. 

Data was collected with self-reported surveys distributed in person in the dialysis unit. The study collection box was kept in an area that was visible for patients at the entrance of the dialysis unit to drop their questionnaires after completion. The participants were provided sufficient time to fill the questionnaire so they could take it home and complete it by the next dialysis session. 

### 2.4. Ethics Considerations

Ethics approval was granted by the hospital’s ethics board (Reference no 471-19).

### 2.5. Data Analysis

Descriptive statistical analyses were performed using SPSS (version 25; SPSS, Chicago, IL, USA). Data were checked for any missing values or outliers. There were no outliers identified. There were few missing values (less than 2%), and those were imputed using the mean of the responses. A *p*-value less than or equal to 0.05 was considered statistically significant. Central tendency measures and frequencies were used to describe the distributions of patients’ demographic and clinical data. Participants’ perceived self-efficacy was determined by calculating the total and mean scores. Pearson’s correlation, Student’s *t*-test, and one-way ANOVA were used to determine the associations of the CKD-SE scores and subscores with the patient characteristics. Cronbach’s alpha was used for the overall CKD-SE score and subscores. A Cronbach’s alpha coefficient greater than 0.70 was considered acceptable.

## 3. Results

### 3.1. Demographic and Disease Characteristics

A total of 190 patients met the inclusion criteria. The demographic characteristics of the participants are described in Table 1. The mean age of patients in the study was 49.24 (SD ± 13.15) years. Approximately half of the participants were males (48.4%), and 75.3% were married. Only 19.5% of the adults were currently working. The majority of the patients (158 out of 190) were undergoing hemodialysis, while the remaining 32 were undergoing peritoneal dialysis. In addition, 36.3% reported having been on dialysis for 1–4 years, and 33.7% had been on dialysis for a longer period (5–10 years). The mean BMI was 28.69 (SD ± 6.7) kg/m^2^, which fell into the overweight category.

### 3.2. Self-Efficacy Findings

The Cronbach’s alpha for the internal consistency and reliability of the total CKD-SE scale was 0.941 and ranged between 0.843 and 0.901 for subscales. The mean self-efficacy score was 192.57 ± 39.23. Table 2 shows the mean score for each subscale: autonomy (62.0 ± 12.8), self-integration (53.76 ± 12.60), problem-solving (46.25 ± 9.94), and seeking social support (30.55 ± 7.18).

Associations of the total score on the CKD-SE and the score on each subscale with the sample characteristics are shown in Table 3. The results show that occupational status and type of dialysis were significantly positively correlated with the perceived self-efficacy score. Additional analysis showed that patients undergoing peritoneal dialysis and patients who were currently working had higher mean total self-efficacy scores and subscale scores.

## 4. Discussion

In the management of chronic diseases, the patient should be involved as a core member of the care team. The self-efficacy of patients undergoing dialysis is a valuable determinant of effective management, nursing interventions, and better outcomes. This study provides primary evidence of the perceived self-efficacy among CKD patients undergoing dialysis in Saudi Arabia. The overall self-efficacy score was 192.57 ± 39.23 among patients on dialysis, which means that the level of self-efficacy was 76.8%. This is a moderate level of self-efficacy, which aligns with similar previous studies conducted with CKD patients [23,24,25]. Evidence shows that patients on dialysis who have better self-efficacy reported better outcomes than those with worse self-efficacy. Additionally, self-efficacy was found to be a mediator of the relationship between knowledge and self-care in CKD patients [21]. However, the efforts have often been centered on patients receiving hemodialysis and have not sought to analyze self-efficacy in those patients who are receiving different types of dialysis [26,27].

In this study, there were no significant associations between age or sex and the self-efficacy score; however, some studies found that older age is associated with a reduced score due to biopsychosocial issues and physiological changes [28]. Overall, the participants in our sample were younger than those in other reported studies, which could explain our results. Frequent reassessment of self-efficacy among dialysis patients in Saudi Arabia is needed due to the gradual shift in the mean age of the population on dialysis in Saudi Arabia.

A significant association was also observed between employment status and perceived disease-related self-efficacy. This result is in accordance with the findings of previous studies [29,30,31] and may be attributable to the relatively higher socioeconomic status of those who are employed, which indicates the availability of more financial resources and a more stable social situation. In addition, previous research has shown that there are positive correlations among empowerment, self-care ability, and self-efficacy. Higher self-efficacy levels are correlated with higher degrees of independence in decision-making in CKD patients [27].

In the current study, the type of dialysis was strongly correlated with the autonomy subscale, and a previous study reported that autonomy was a key dimension of self-efficacy and disease control in patients on long-term peritoneal dialysis [32]. Interestingly, the type of dialysis was significantly correlated with patient self-efficacy; patients on peritoneal dialysis had greater perceived disease-related self-efficacy than those undergoing hemodialysis. This finding aligns with another study in which it was hypothesized that receiving a home dialysis modality such as peritoneal dialysis contributes to greater self-efficacy than receiving a traditional in-center modality such as hemodialysis [33]. It is thought that patients undergoing peritoneal dialysis are more independent because most of them are managing peritoneal dialysis by themselves. Another study showed that patients undergoing peritoneal dialysis had higher scores for positive attitude, stress reduction, and decision making than did hemodialysis patients [27]. This finding indicates that more attention should be given to patients undergoing hemodialysis.

### 4.1. Implications for Clinical Practice

Living with ESKD requires patients to actively manage their disease. Effective self-management is associated with a higher level of self-efficacy. Nurses provide critical care, such as dialysis. In addition, they are also responsible for providing education to the patients and empowering patient self-care behavior. All healthcare professionals, including nurses, should focus on the early identification of patients with low levels of perceived self-efficacy and develop individualized treatment interventions. More attention should be given to implementing effective strategies to improve self-efficacy among patients on hemodialysis. Further research on this topic would aid in delivering patient-centered behavioral methods to support patient care and ultimately improve health outcomes.

### 4.2. Limitations

As it was a cross-sectional study, the dynamic changes over time were not investigated. The results may not be generalized because of the small sample size and limited sampling selection. There may have been information bias due to the use of a self-reported measure.

## 5. Conclusions

The results of the current study show that greater self-efficacy was associated with employment and the use of peritoneal dialysis. However, further research using a larger sample size to assess the overall perceived self-efficacy in dialysis patients and the associated risk factors is still needed. Moreover, strategies to enhance self-efficacy among dialysis patients, especially those on hemodialysis, are needed.

## Figures and Tables

**Table 1:** Characteristics of the study population, *n* = 190.

Variable	*n*	%	Mean	SD
**Age**			49.24	13.15
**Gender**				
Male	92	48.4		
Female	98	51.6		
**Marital status**				
Unmarried	47	24.7		
Married	143	75.3		
**Education level**				
Illiterate	38	20.0		
High School or less	107	56.3		
Bachelor degree	43	22.6		
Postgraduate	2	1.1		
**Occupational status**				
Unemployed	153	80.5		
Employee	37	19.5		
**Type of dialysis therapy**				
Hemodialysis	158	83.2		
Peritoneal dialysis	32	16.8		
**Time using dialysis therapy**				
0–12 months (less than a year)	26	13.7		
1–4 years	69	36.3		
5–10 years	64	33.7		
More than 10 years	31	16.3		
**Body mass index**			28.69	6.7
**Blood pressure (mmHg)**				
Systolic			142.87	22.5
Diastolic			79.92	13.42

Note: SD = standard deviation.

**Table 2:** Descriptive statistics and Cronbach’s alpha of the CKD-SE scale.

Subscale	Potential Range	Actual Range	*M* (*SD*)	Cronbach’s Alpha
Autonomy	8–80	25–80	62.00 (12.81)	0.826
Self-integration	7–70	16–70	53.76 (12.60)	0.897
Problem solving	6–60	15–60	46.25 (9.94)	0.823
Seeking social support	4–40	10–40	30.55 (7.18)	0.741
Total score for CKD-SE	25–250	81–250	192.57 (39.23)	0.921

Note: CKD-SE = chronic kidney disease self-efficacy.

**Table 3:** Correlation between Perceived Self-efficacy and sample characteristic.

Variable	Autonomy	Self-Integration	Problem Solving	Seeking Social Support	Total Score for CKD-SE
Age †	−0.028	−0.03	−0.13	−0.005	−0.05
Gender ‡	−1.19	−0.55	−0.76	−1.33	−1.007
Marital status ‡	−1.38	−1.4	−0.77	−1.22	−1.34
Education level §	0.761	1.49	2.33 *	0.47	1.30
Occupational status ‡	2.44 *	2.12 *	2.86 **	1.52 *	2.48 *
Type of dialysis therapy ‡	2.83 **	2.84 **	2.48 *	2.89 **	3.00 **
Time using dialysis therapy §	0.33	1.01	0.63	2.62 **	0.95
Body mass index †	0.05	0.03	0.068	0.062	0.058

** Correlation is significant at *p* < 0.01; * Correlation is significant at *p* < 0.05; † Pearson Correlation test; ‡ *t*-test; § One-way ANOVA test.

## Data Availability

Availability of data and materials: All data generated or analyzed during this study are included in this published article.
